# Non-linear association between thyroid scintigraphy-derived thyroid weight and I-131 treatment efficacy in graves’ disease: a multicenter restricted cubic spline and threshold analysis

**DOI:** 10.3389/fendo.2026.1802163

**Published:** 2026-07-17

**Authors:** Lu Lu, LeLe Zhang, Dongyun Meng, Xiaojuan Wei, Yan Chen, Shaozhou Mo, Zeyong Sun, Fengyang Song, Yuehua Li, Xingyu Mu, Wentan Huang, Wei Fu, Kehua Liao

**Affiliations:** 1 Department of Nuclear Medicine, The People’s Hospital of Guangxi Zhuang Autonomous Region, Nanning, Guangxi Zhuang, China; 2 Department of Nuclear Medicine, First Affiliated Hospital of Guilin Medical University, Guilin, Guangxi Zhuang, China

**Keywords:** graves’ disease, iodine-131, multi-center study, restricted cubic splines, threshold effect, thyroid weight

## Abstract

**Objective:**

Radioactive iodine-131 (I-131) is a standard therapy for Graves’ disease (GD), yet predicting therapeutic efficacy remains challenging due to complex biological variables. Conventional linear models often fail to capture the dynamic dose-response relationship between thyroid size and treatment outcomes. This study aimed to characterize the non-linear relationship between thyroid weight and I-131 efficacy and identify critical clinical thresholds influencing therapeutic success.

**Methods:**

A retrospective multicenter cohort study was conducted involving 888 records, of whom 612 GD patients received initial I-131 treatment between 2018 and 2024. Thyroid weight was estimated using thyroid scintigraphy. Restricted cubic splines (RCS) were employed to model potential non-linear associations, and piecewise threshold analysis was utilized to determine optimal inflection points regarding the correlation between thyroid weight and treatment non-remission.

**Results:**

Among 612 patients (mean age 41.2 ± 12.4 years; 73.9% female), 250 (40.8%) experienced non-remission after initial I-131 therapy. RCS analysis revealed a significant non-linear association between thyroid weight and treatment outcome in both unadjusted (P-nonlinear = 0.034) and DAG-confounder-adjusted models including study center (P-nonlinear = 0.032). In multivariable DAG-based logistic regression, thyroid weight (adjusted OR = 1.025, 95% CI: 1.017-1.034), female gender (adjusted OR = 1.67, 95% CI: 1.09-2.56), disease duration >2 years (adjusted OR = 1.97, 95% CI: 1.30-2.99), and study center (adjusted OR = 0.62, 95% CI: 0.42-0.93) were independent predictors. Piecewise analysis identified an exploratory inflection point at 46 g (adjusted LRT: P = 0.030). Mediation analysis demonstrated that RAIU at 3 h was a modest partial mediator (Sobel Z = 2.43, P = 0.015; mediated proportion: 5.8%).

**Conclusion:**

Thyroid weight demonstrates a significant non-linear association with I-131 treatment efficacy in GD, with an exploratory inflection point at approximately 46 g. The association is robust to DAG-based confounder adjustment including study center and is only modestly mediated by radioiodine uptake. These findings support individualized RAI dosing strategies incorporating thyroid weight thresholds.

## 1 Introduction

Graves’ disease (GD) is the leading cause of autoimmune hyperthyroidism, particularly in regions with adequate iodine intake (1, 2). The condition is driven by thyroid-stimulating hormone receptor antibodies (TRAb), which bind to TSH receptors on follicular cells. This interaction induces excessive thyroid stimulation, resulting in goiter and the overproduction of thyroid hormones (T3 and T4) (2). Although GD can manifest at any age, including in children and adolescents, it is most frequently observed in females (3). If left untreated, the disease can cause long-term detriment to cardiovascular health, bone integrity, and mental well-being.

Three primary treatment modalities are established for GD: antithyroid drugs (ATD), radioactive iodine-131 (I-131) therapy, and thyroidectomy (4). Although ATD administered over 12–18 months is typically the first-line treatment, it is associated with high relapse rates following discontinuation (5). I-131 therapy therefore serves as an effective definitive treatment, achieving success rates exceeding 90% (6).

In I-131 therapy, thyroid weight is the central parameter for individualized dosimetry, directly determining the administered activity through the standard dose formula (4, 7). Accurate thyroid weight estimation is essential for optimizing the balance between therapeutic efficacy and the risk of post-treatment hypothyroidism. Thyroid scintigraphy with ^99m^TcO_4_^-^ provides both functional imaging and an estimate of thyroid weight as part of the standard pre-therapeutic workup (7, 8).

Despite the central role of thyroid weight in dose determination, the precise nature of its relationship with I-131 treatment outcome remains incompletely characterized. Current evidence indicates an inverse association: larger glands are consistently associated with higher failure rates (9, 10), while smaller glands carry an elevated risk of post-treatment hypothyroidism (11). However, whether this relationship follows a simple linear gradient or exhibits more complex, non-linear dynamics has not been systematically evaluated.

The relationship between thyroid weight and RAI treatment outcome is inherently complex and plausibly non-linear. Thyroid weight directly influences the administered I-131 activity through the dose calculation formula, yet the treatment outcome is determined not solely by administered activity but also by intra-thyroidal dose distribution, tissue radiosensitivity, and the biological effective half-life. Restricted cubic spline (RCS) analysis imposes no *a priori* assumption of linearity and can detect non-linear patterns including potential inflection points (12). Coupled with piecewise threshold analysis (13, 14), this framework enables granular characterization of dose-response relationships. Identifying thyroid weight thresholds may have direct clinical implications for individualized RAI dose planning in GD.

This study aimed to characterize the non-linear relationship between thyroid weight and I-131 treatment outcome in patients with GD using RCS and piecewise threshold analysis, and to identify clinically relevant thyroid weight thresholds for individualized treatment planning.

## 2 Methods

### 2.1 Study design and population

This retrospective cohort study screened 888 patient records from two nuclear medicine centers. After applying inclusion and exclusion criteria, 612 patients with GD who received initial I-131 therapy were included. The cohort comprised 321 patients treated at the Department of Nuclear Medicine, The People’s Hospital of Guangxi Zhuang Autonomous Region (Center 1) between June 2018 and July 2024, and 291 patients treated at the Department of Nuclear Medicine, First Affiliated Hospital of Guilin Medical University (Center 2) between October 2018 and September 2023. The diagnosis of GD was based on the 2016 American Thyroid Association guideline (15).

Inclusion criteria were: (i) confirmed GD with clinical symptoms, elevated FT3 and FT4, suppressed TSH, and positive TRAb; (ii) first-time I-131 therapy; (iii) pretreatment thyroid scintigraphy available; and (iv) complete follow-up data. Exclusion criteria were: (i) other thyroid disorders (toxic multinodular goiter, toxic adenoma, thyroiditis, malignancy); (ii) prior thyroid surgery or cervical irradiation; (iii) incomplete essential data; (iv) pregnancy or lactation; and (v) severe systemic comorbidities.

**Figure 1:**
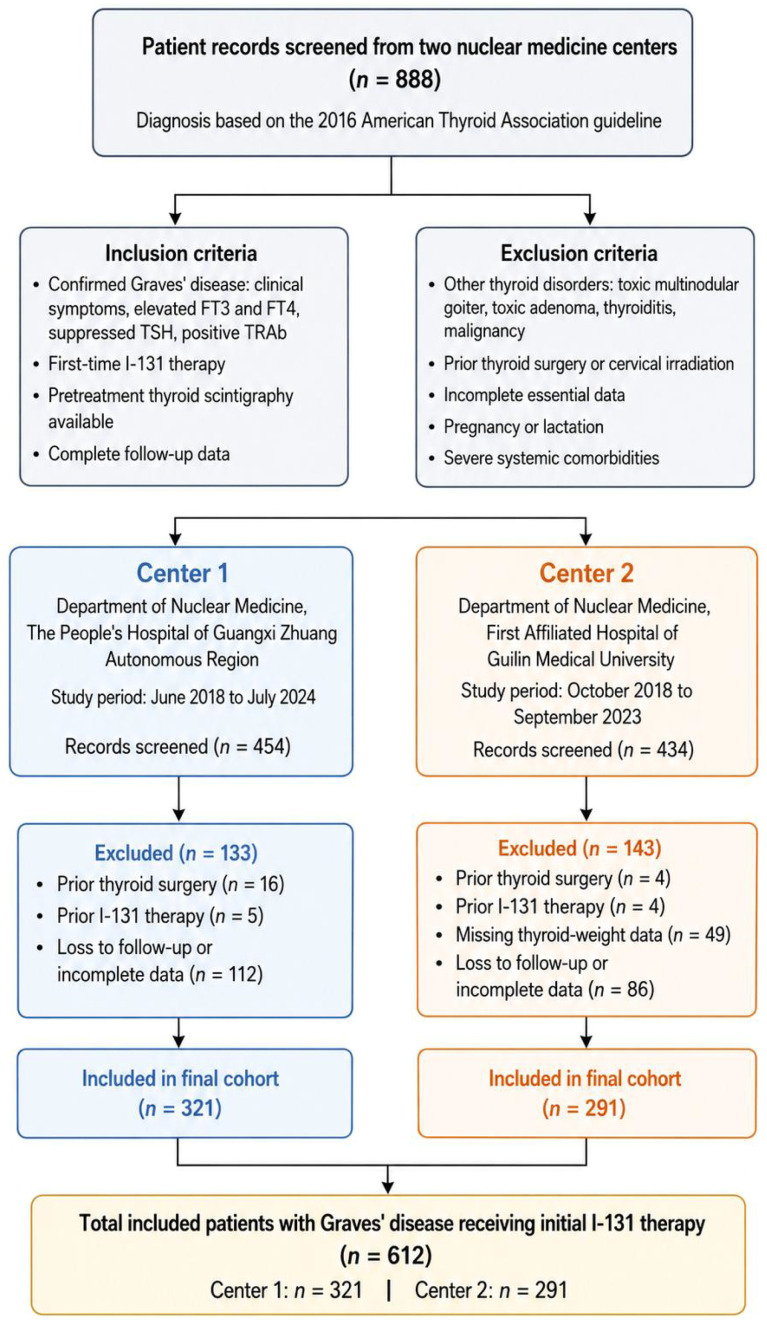
Patient flow diagram.

At Center 1, a total of 454 records were reviewed, resulting in the exclusion of 133 cases due to prior thyroid surgery (n = 16), previous I-131 therapy (n = 5), and loss to follow-up or incomplete data (n = 112). Similarly, at Center 2, 434 records were assessed, with 143 exclusions attributed to prior thyroid surgery (n = 4), previous I-131 therapy (n = 4), missing thyroid-weight data (n = 49), and loss to follow-up or incomplete data (n = 86), as illustrated in **Figure 1**.

### 2.2 Data collection

Clinical data were extracted from electronic medical records and imaging archives. Key variables included demographics (gender and age at initial therapy). Thyroid hormones and antibodies (TSH, T3, T4, FT3, FT4, TPOAb, and TRAb) were quantified via chemiluminescence immunoassay. Center 1 utilized the UniCel DxI 800 Access Immunoassay System, while Center 2 employed the Roche cobas e801 fully automated immunoassay analyzer.

Radioactive iodine uptake (RAIU) was assessed using I-131 sourced from Nanning Atomic High-throughput Isotope Co., Ltd. Patients abstained from iodine-rich foods and medications for 2–4 weeks prior to testing. On the test day, fasting patients ingested 2-10 μCi of sodium I-131. Thyroid radioactivity was measured at 3 and 24 hours post-administration using the NM-6110 instrument at Center 1 and the JG-2000 thyroid function analyzer (Xi’an Kaipu Electromechanical Co., Ltd.) at Center 2. The effective half-life (Teff) was calculated from these values.

Thyroid weight was determined via planar scintigraphy performed 15–20 minutes after intravenous injection of 2-5 mCi of ^99m^TcO_4_^-^. Patients were positioned supine with neck extension. At Center 1, images were acquired using a Discovery NM/CT 670 system (GE Healthcare); at Center 2, a Symbia T16 SPECT/CT system (Siemens Healthineers) was used. Thyroid volume was quantified by region-of-interest (ROI) analysis (Xeleris at Center 1; syngo.via at Center 2). A standardized protocol was applied: the thyroid ROI was defined by a 20% isocontour threshold of the maximum pixel count, with background subtraction. Thyroid weight was calculated using the geometric mean method, assuming a tissue density of 1.0 g/mL. All measurements were independently reviewed by a second nuclear medicine physician.

History of antithyroid drug (ATD) therapy was recorded to categorize patients as either ATD-naïve or ATD-experienced.

Disease duration was stratified as ≤2 years or >2 years.

### 2.3 I-131 treatment dose

Patients were instructed to adhere to a low-iodine diet and avoid iodide-containing medications for 7–14 days before treatment. ATD therapy was discontinued at least one week prior to I-131 administration. The treatment dose was calculated using the formula: *Dose (μCi) = [Dose per gram of thyroid tissue (μCi/g) × Thyroid weight (g)]/24-hour thyroid uptake (%).* Doses were administered via a fully automated dispensing system. Three experienced nuclear medicine physicians prescribed the iodine dose per gram (IDPG) based on clinical assessment. For analysis, IDPG was categorized as low (70–90 μCi/g) or high (91–120 μCi/g).

### 2.4 Assessment of therapeutic efficacy

Therapeutic efficacy was initially evaluated 4–8 weeks post-RAI therapy. Thyroid function was monitored every 4–8 weeks for up to 12 months. Treatment outcomes were categorized into four groups: (1) complete remission; (2) hypothyroidism; (3) partial remission; and (4) ineffective response. Complete remission or hypothyroidism were grouped as Remission (n = 362, 59.2%), and partial remission or ineffective response as Non-Remission (n = 250, 40.8%). The four-category distribution was: complete remission, n = 71 (11.6%); hypothyroidism, n = 291 (47.5%); partial remission, n = 204 (33.3%); ineffective response, n = 46 (7.5%).

### 2.5 Statistical analysis

**Figure 2:**
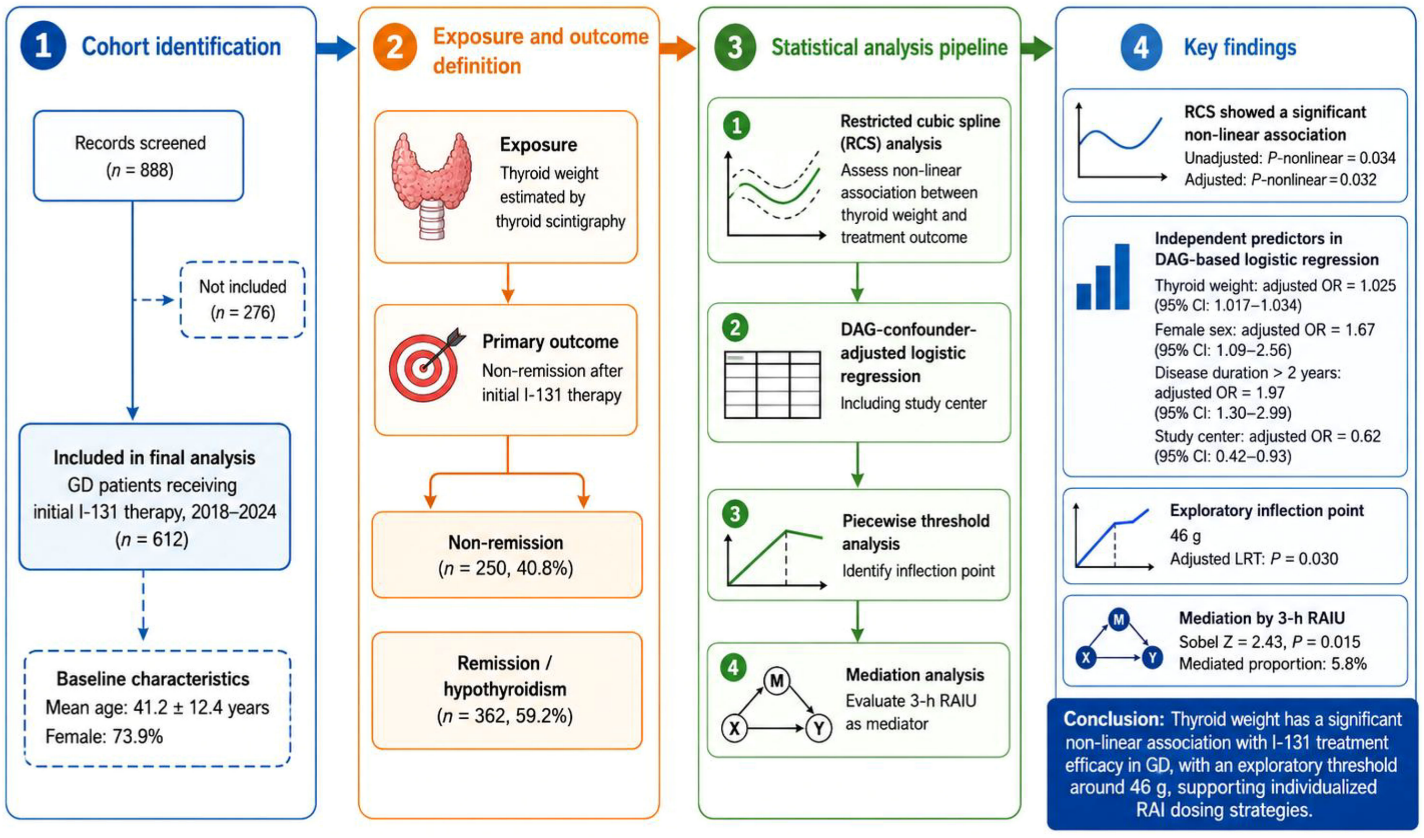
Workflow.

Statistical analyses were performed using R version 4.2.2 (R Foundation for Statistical Computing, Vienna, Austria) with the rms (v6.7-0), car (v3.1-1), and MASS (v7.3-58) packages. Categorical variables were presented as frequencies [n (%)] and compared using the chi-square test. Continuous variables were presented as mean ± SD or median (interquartile range) and compared using the independent-samples t-test or the Mann-Whitney U test, as appropriate. Covariate selection followed a Directed Acyclic Graph (DAG)-based causal framework (**Supplementary Figure 1**), with variables classified as confounders (age, gender, ATD history, disease duration >2 years, FT3, TPOAB, TRAB, and study center) or mediators (TID, IDPG, RAIU3h, RAIU24h, Teff; **Supplementary Table 1**). Confounders were forced into all multivariable models; mediators were excluded from the primary model to avoid overadjustment bias. FT4 and RAIU24h were excluded due to high collinearity with FT3 (r = 0.85) and RAIU3h (r = 0.78), respectively. Three pre-specified logistic regression models were constructed: Model 1 (total effect) included all eight confounders; Model 2 (direct effect) additionally included RAIU3h; and Model 3 (adjusted RCS) applied restricted cubic splines to thyroid weight with three knots at the 10th, 50th, and 90th percentiles, adjusted for the Model 1 confounder set. The overall study workflow is shown in **Figure 2**. Multicollinearity was assessed using variance inflation factors (VIF >5: moderate; >10: severe; **Supplementary Table 2**). Non-linearity of the RCS model was tested by the Wald test on the nonlinear spline coefficient and by the likelihood ratio test comparing the RCS model to the linear specification. Piecewise logistic regression was performed with the inflection point set at 46 g, identified from the RCS curve. Mediation by RAIU3h was assessed using the Baron-Kenny causal-steps framework with the Sobel test. Sensitivity analyses included a change-in-estimate evaluation (**Supplementary Table 3**), a female-only subgroup analysis, and analyses with alternative outcome definitions. A two-sided P < 0.05 was considered statistically significant.

## 3 Results

### 3.1 Patient characteristics

**Table 1** summarizes the baseline characteristics for the study cohorts from Center 1 (n=321) and Center 2 (n=291), stratified by treatment outcome (remission vs. non-remission). In the Center 1 cohort, significant differences between outcome groups were observed in gender (P = 0.003), history of ATD use (P = 0.003), disease duration >2 years (P = 0.001), TID (P<0.001), TRAb levels (P = 0.006), RAIU at 3 hours (P<0.001) and 24 hours (P = 0.017), Teff (P<0.001), and thyroid weight (P<0.001). No significant differences were found for age, FT3, FT4, TPOAb, or IDPG. In contrast, the Center 2 cohort exhibited significant differences in age (P = 0.031), FT3 (P = 0.039), FT4 (P = 0.004), RAIU at 3 hours (P<0.001) and 24 hours (P = 0.011), Teff (P = 0.041), thyroid weight (P<0.001), and IDPG (P = 0.019). Variables such as gender, history of ATD use, disease duration >2 years, TID, TPOAb, and TRAb did not differ significantly between outcome groups in this cohort.

**Table 1:** Patient demographics and baseline characteristics.

Characteristic	Center 1, N = 321				Center 2, N = 291			
	Overall N = 321	Remission N = 161	Non-Remission N = 160	p-value^1^	Overall N = 291	Remission N = 201	Non-Remission N = 90	p-value^1^
Gender, n (%)				0.003				0.161
male	79 (24.6%)	51 (31.7%)	28 (17.5%)		81 (27.8%)	51 (25.4%)	30 (33.3%)	
female	242 (75.4%)	110 (68.3%)	132 (82.5%)		210 (72.2%)	150 (74.6%)	60 (66.7%)	
Age, Median (Q1, Q3)	37 (27, 44)	37 (28, 45)	37 (27, 44)	0.743	43 (32, 51)	44 (33, 52)	42 (30, 48)	0.031
the_history_of_ATD_usage, n (%)				0.003				0.071
No	63 (19.6%)	42 (26.1%)	21 (13.1%)		96 (33.0%)	73 (36.3%)	23 (25.6%)	
Yes	258 (80.4%)	119 (73.9%)	139 (86.9%)		195 (67.0%)	128 (63.7%)	67 (74.4%)	
Disease_course_over_2years, n (%)				0.001				<0.001
No	223 (69.5%)	125 (77.6%)	98 (61.3%)		185 (63.6%)	144 (71.6%)	41 (45.6%)	
Yes	98 (30.5%)	36 (22.4%)	62 (38.8%)		106 (36.4%)	57 (28.4%)	49 (54.4%)	
TID, Median (Q1, Q3)	5.5 (4.5, 8.0)	5.0 (4.2, 6.0)	6.5 (5.0, 10.0)	<0.001	7.9 (6.2, 10.5)	7.8 (6.0, 9.8)	8.6 (6.3, 12.0)	0.040
FT3, Median (Q1, Q3)	22 (12, 33)	22 (14, 31)	20 (11, 34)	0.620	26 (17, 37)	26 (17, 35)	29 (19, 39)	0.039
FT4, Median (Q1, Q3)	53 (35, 65)	54 (41, 64)	50 (32, 68)	0.603	65 (45, 100)	62 (43, 92)	83 (52, 100)	0.004
TPOAB, Median (Q1, Q3)	287 (58, 879)	279 (55, 726)	307 (59, 960)	0.252	212 (86, 397)	225 (98, 431)	200 (42, 345)	0.116
TRAB, Median (Q1, Q3)	17 (10, 29)	15 (9, 23)	20 (10, 34)	0.006	15 (7, 24)	15 (7, 23)	14 (6, 24)	0.792
RAIU3h, Median (Q1, Q3)	74 (56, 87)	67 (52, 81)	80 (64, 92)	<0.001	57 (43, 70)	53 (41, 67)	63 (51, 74)	<0.001
RAIU24h, Median (Q1, Q3)	87 (77, 95)	86 (74, 93)	89 (79, 96)	0.017	65 (53, 75)	64 (53, 73)	68 (58, 78)	0.011
Teff, Median (Q1, Q3)	5.33 (4.80, 5.95)	5.60 (4.96, 6.12)	5.04 (4.62, 5.67)	<0.001	5.24 (4.68, 5.73)	5.29 (4.80, 5.79)	5.11 (4.59, 5.55)	0.041
Thyroid_weight, Median (Q1, Q3)	55 (40, 78)	47 (36, 60)	65 (47, 92)	<0.001	39 (31, 55)	37 (27, 52)	45 (36, 67)	<0.001
IDPG, n (%)				0.868				0.019
70–90 μCi/g	155 (48.3%)	77 (47.8%)	78 (48.8%)		46 (15.8%)	25 (12.4%)	21 (23.3%)	
91–120 μCi/g	166 (51.7%)	84 (52.2%)	82 (51.3%)		245 (84.2%)	176 (87.6%)	69 (76.7%)	

^1^ Pearson’s Chi-squared test; Wilcoxon rank sum test.

### 3.2 Correlation analysis between thyroid weight and therapeutic outcomes

Correlation analysis was conducted on the total sample of 612 patients to evaluate the relationship between thyroid weight and therapeutic efficacy (**Table 2**). Using point-biserial correlation, a statistically significant negative correlation was observed between thyroid weight and clinical remission (rho = -0.340; 95% CI: -0.408 to -0.268; P < 0.001). This inverse relationship indicates that higher thyroid weight is associated with a reduced likelihood of achieving remission. **Supplementary Figure 2** displays the correlation matrix summarizing these interactions, confirming that increased thyroid burden serves as a significant negative predictor for treatment success.

**Table 2:** Correlation analysis results.

Parameter1	Parameter2	rho	CI	CI_low	CI_high	t	df_error	p	Method	n_Obs
Thyroid weight	Remission	-0.3401242	0.95	-0.4083711	-0.2680960	-8.933031	610	<0.001	Point-biserial correlation	612
Thyroid weight	Non-remission	0.3401242	0.95	0.2680960	0.4083711	8.933031	610	<0.001	Point-biserial correlation	612

### 3.3 Univariate and multivariate logistic regression analyses

**Figure 3:**
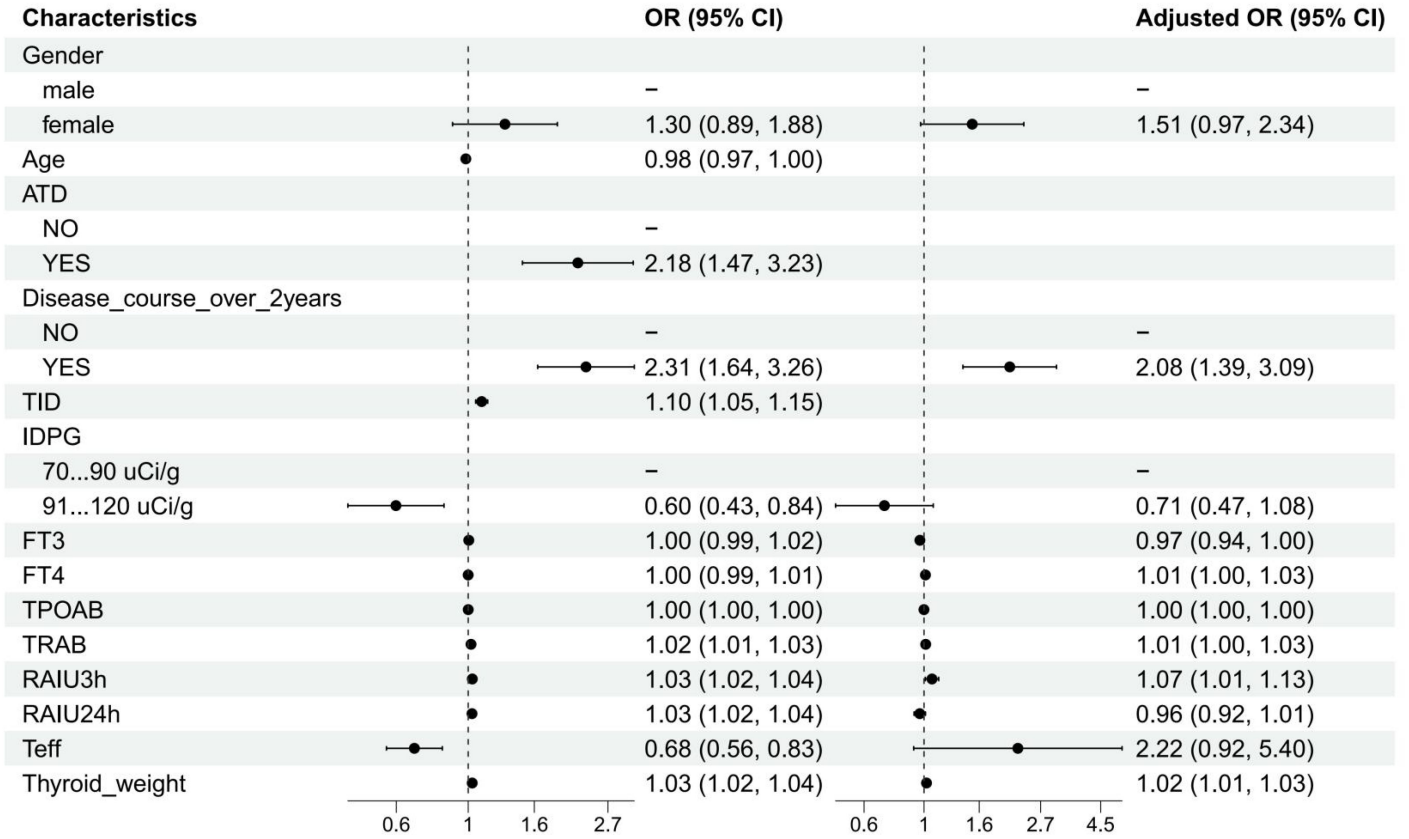
Univariate and multivariate analysis forest plot.

Multivariable logistic regression using the DAG-based confounder set (Model 1: age, gender, ATD history, disease duration >2 years, FT3, TPOAB, TRAB, and study center) identified thyroid weight as an independent predictor of non-remission (adjusted OR = 1.025 per gram, 95% CI: 1.017–1.034, P < 0.001; **Table 3**). Female gender was associated with a 67% higher odds of non-remission (adjusted OR = 1.67, 95% CI: 1.09–2.56, P = 0.019). Disease duration exceeding two years independently predicted non-remission (adjusted OR = 1.97, 95% CI: 1.30–2.99, P = 0.001). Study center was a significant independent predictor: patients treated at Center 2 had approximately 38% lower odds of non-remission than those treated at Center 1 (adjusted OR = 0.62, 95% CI: 0.42-0.93, P = 0.022). Age, ATD history, FT3, TPOAB, and TRAB were not significant independent predictors in the DAG-adjusted model (all P > 0.05). Multicollinearity was negligible (all VIF < 1.33). The model AUC was 0.742 (95% CI: 0.702–0.782). When RAIU3h was added to assess the direct effect (Model 2), the thyroid weight OR attenuated modestly to 1.023 (95% CI: 1.014–1.031, P < 0.001), representing an approximately 11% attenuation of the total effect. The univariate and multivariable results for all variables are summarized in **Table 3**, **Figure 3**.

**Table 3:** Univariate and multivariable analysis of predictors of non-remission (DAG-based Model 1 and Model 2).

Characteristic	Univariable					Multivariable				
	N	Event N	OR	95% CI	p-value	N	Event N	OR	95% CI	p-value
Gender										
male	160	58	—	—		160	58	1.67	1.09–2.56	0.019
female	452	192	1.30	0.89, 1.88	0.169	452	192	1.67	1.09-2.56	0.019
Age	612	250	0.98	0.97, 1.00	0.009			1.000	0.986-1.015	0.988
the_history_of_ATD_usage										
NO	159	44	—	—						
YES	453	206	2.18	1.47, 3.23	<0.001			1.255	0.801-1.967	0.322
Disease_course_over_2years										
NO	408	139	—	—		408	139	1.97	1.30–2.99	0.001
YES	204	111	2.31	1.64, 3.26	<0.001	204	111	1.97	1.30-2.99	0.001
TID	612	250	1.10	1.05, 1.15	<0.001					
IDPG										
70–90 µCi/g	201	99	—	—		201	99	—	—	
91–120 µCi/g	411	151	0.60	0.43, 0.84	0.003	411	151	0.71	0.47, 1.08	0.112
FT3	612	250	1.00	0.99, 1.02	0.521	612	250	0.989	0.974-1.004	0.164
FT4	612	250	1.00	0.99, 1.01	0.942	612	250	1.01	1.00, 1.03	0.113
TPOAB	612	250	1.00	1.00, 1.00	0.215	612	250	1.000	0.999-1.001	0.295
TRAB	612	250	1.02	1.01, 1.03	0.004	612	250	1.014	0.995-1.033	0.145
RAIU3h	612	250	1.03	1.02, 1.04	<0.001	612	250	1.07	1.01, 1.13	0.020
RAIU24h	612	250	1.03	1.02, 1.04	<0.001	612	250	0.96	0.92, 1.01	0.144
Teff	612	250	0.68	0.56, 0.83	<0.001	612	250	2.22	0.92, 5.40	0.077
Thyroid_weight	612	250	1.03	1.02, 1.04	<0.001	612	250	1.025	1.017-1.034	<0.001

CI, Confidence Interval; OR, Odds Ratio.

Null deviance, 828; Null df, 611; Log-likelihood, -350; AIC, 724; BIC, 777; Deviance, 700; Residual df, 600; No. Obs., 612.

### 3.4 RCS analysis

**Figure 4:**
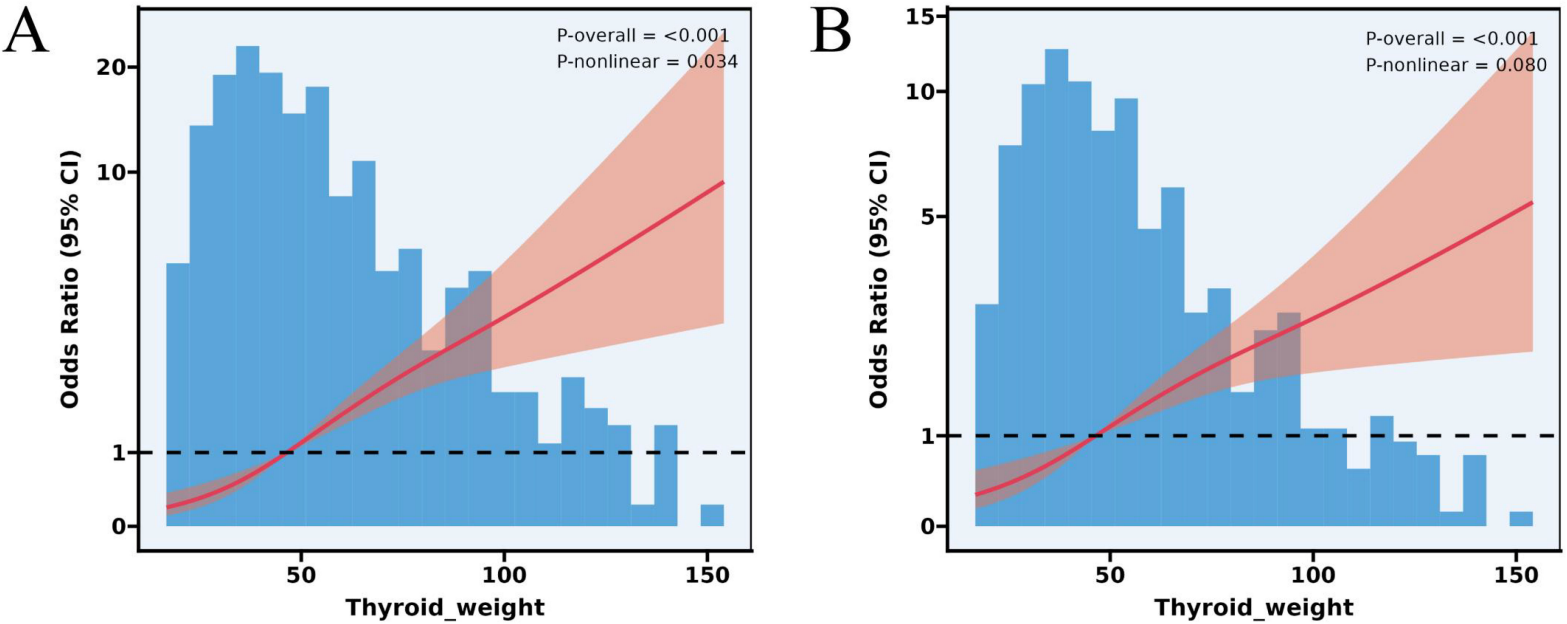
RCS analysis of the association between thyroid weight and treatment outcome. **(A)** Unadjusted RCS plot illustrating the relationship between thyroid weight and the odds ratio. The association was statistically significant (P-overall<0.001), with a confirmed significant non-linear relationship (P-nonlinear =0.034). **(B)** Adjusted RCS plot derived from a logistic regression model adjusted for DAG-specified confounders: age, gender, ATD history, disease duration >2 years, FT3, TPOAB, TRAB, and study center. The overall association remained significant (P-overall<0.001), confirming the non-linear association was statistically significant (P-nonlinear=0.032). In both panels, the solid red line represents the estimated odds ratio, and the pink shaded area indicates the 95% confidence interval. The blue histogram in the background depicts the distribution of thyroid weight. The black dashed horizontal line represents the reference odds ratio of 1.0.

RCS analysis with three knots (10th, 50th, and 90th percentiles: 26.6 g, 46.5 g, and 95.1 g) was employed to characterize the dose–response relationship between thyroid weight and treatment non-remission (**Figure 4**). In the unadjusted model, the association between thyroid weight and the odds of non-remission was statistically significant (P-overall < 0.001), and the likelihood ratio test confirmed a significant departure from linearity (P-nonlinear = 0.034). The unadjusted RCS curve demonstrated a gradual increase in the odds of non-remission across the thyroid weight spectrum (20–120 g), with a steeper incline between approximately 40 g and 80 g and a modest attenuation beyond 80 g.

In the fully adjusted RCS model (Model 3), which incorporated the identical DAG confounder set as Model 1 (age, gender, ATD history, disease duration >2 years, FT3, TPOAB, TRAB, and study center), the overall association between thyroid weight and non-remission remained highly significant (P-overall < 0.001). The non-linear component was statistically significant (Wald test: P-nonlinear = 0.032; likelihood ratio test comparing RCS to linear: χ² = 4.38, P = 0.036), confirming that the dose–response relationship is better characterized by a non-linear than a linear function after adjustment for all DAG-specified confounders. Compared with the original covariate specification (which included the mediator RAIU3h and yielded P-nonlinear = 0.080), the DAG-based confounder set reveals the non-linearity more clearly, consistent with the expectation that adjusting for mediators attenuates the exposure–outcome association (overadjustment bias). The AIC values supported the non-linear specification (RCS AIC = 733.0 vs. linear AIC = 735.4; ΔAIC = 2.4).

### 3.5 Piecewise threshold analysis

**Figure 5:**
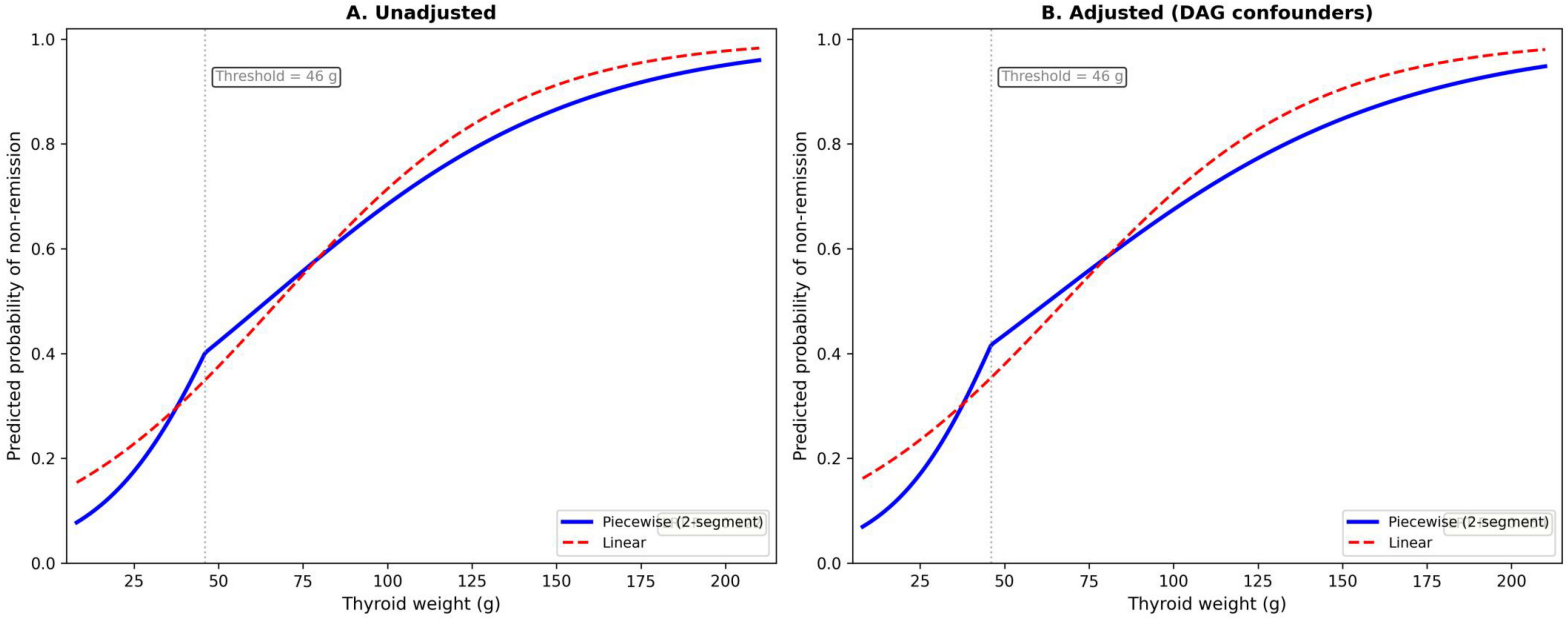
Piecewise threshold analysis of thyroid weight on treatment outcome. **(A)** Unadjusted piecewise regression model. **(B)** Adjusted piecewise regression model (DAG Model 1 confounders including study center). In both panels, the solid blue line represents the piecewise model fit, the dashed red line represents the linear model, and the shaded area indicates the 95% confidence band. The rug plot along the x-axis shows the distribution of thyroid weight.

To further characterize the dose–response relationship and identify potential threshold effects, piecewise logistic regression was performed with the inflection point set at 46 g (**Tables 4**, **5**; **Figure 5**). In the unadjusted analysis, the linear model yielded an OR of 1.03 per gram (95% CI: 1.02–1.04, P < 0.001). The two-segment piecewise model identified a significant inflection at 46 g: below this threshold, each 1-g increase in thyroid weight was associated with a 6% increase in the odds of non-remission (OR = 1.06, 95% CI: 1.03–1.08, P < 0.001), whereas at or above 46 g, the incremental risk was 2% per gram (OR = 1.02, 95% CI: 1.01–1.03, P < 0.001). The log-likelihood ratio test confirmed that the piecewise model provided a significantly better fit than the linear model (LRT: P = 0.040).

**Table 4:** Threshold effect analysis of thyroid weight on treatment outcome.

Model/Variable	OR (95% CI)	P-value
**Fitting by standard Logistic regression model**	1.03 (1.02, 1.04)	<0.001
Fitting by piecewise Logistic regression model (break-point = 46)		
Thyroid_weight < 46	1.06 (1.03, 1.08)	<0.001
Thyroid_weight ≥ 46	1.02 (1.01, 1.03)	<0.001
**Log likelihood ratio**		**0.040**

Bold values indicate statistical significance (P < 0.05).

**Table 5:** Adjusted threshold effect analysis of thyroid weight on treatment outcome (DAG Model 1 confounders including study center).

Model/Variable	OR (95% CI)*	P-value
**Fitting by standard Logistic regression model**	1.02 (1.01, 1.03)	<0.001
Fitting by piecewise Logistic regression model		
Thyroid_weight < 46	1.056 (1.026-1.086)	<0.001
Thyroid_weight ≥ 46	1.075 (1.047-1.105)	<0.001
**Log likelihood ratio**		**0.030**

*Adjusted for DAG confounders: age, gender, ATD history, disease duration >2 yr, FT3, TPOAB, TRAB, and study center.Bold values indicate statistical significance (P < 0.05).

In the adjusted analysis incorporating all DAG confounders including study center (**Table 5**), the inflection point remained at 46 g. The adjusted per-gram odds ratios were 1.056 (95% CI: 1.026–1.086, P < 0.001) below 46 g and 1.075 (95% CI: 1.047–1.105, P < 0.001) at or above 46 g, indicating a mildly accelerating, continuous dose–effect relationship across the thyroid weight range. The likelihood ratio test confirmed that the adjusted piecewise model significantly outperformed the adjusted linear model (LRT: χ² = 4.69, P = 0.030). The AIC values (linear: 735.4; RCS: 733.0; piecewise: 732.7) indicate that both the RCS and piecewise models outperform the linear specification, with the piecewise model showing a marginal advantage. The overlapping confidence intervals of the two segment slopes (1.026–1.086 vs. 1.047–1.105) suggest a continuously increasing, mildly accelerating dose–effect relationship rather than a discrete plateau. The 46-g inflection point should therefore be regarded as an exploratory threshold.

### 3.6 Mediation analysis

Formal mediation analysis assessed whether RAIU3h mediates the thyroid weight-treatment outcome association, adjusted for all DAG confounders including study center. In the Baron-Kenny framework: (a) total effect of thyroid weight was significant (OR = 1.025, P<0.001); (b) thyroid weight was associated with RAIU3h (beta=0.086, P<0.001); (c) RAIU3h predicted non-remission controlling for thyroid weight (OR = 1.018, P = 0.002); (d) direct effect remained significant (OR = 1.023, P<0.001). The Sobel test confirmed a significant indirect effect (Z = 2.43, P = 0.015; mediated proportion: 5.8%), indicating that >94% of the thyroid weight effect operates through pathways independent of radioiodine uptake.

### 3.7 Female subgroup sensitivity analysis

Given the female predominance of GD, a pre-specified subgroup analysis was conducted in the 452 female participants (73.9% of the cohort; 192 non-remission events, 42.5%). In the DAG-adjusted logistic regression model (excluding gender, including study center), each 1-g increase in thyroid weight was associated with a 4.1% increase in the odds of non-remission (adjusted OR = 1.041, 95% CI: 1.028-1.053, P < 0.001). Disease duration >2 years was not a significant independent predictor (adjusted OR = 1.51, 95% CI: 0.91-2.52, P = 0.111). Restricted cubic spline analysis in the female subgroup did not confirm a non-linear association (P-nonlinear = 0.813; LRT: chi2 = 0.06, P = 0.814), indicating that a linear model adequately describes the thyroid weight-outcome relationship in female GD patients. This finding suggests that the overall non-linearity detected in the full cohort may partially reflect the greater thyroid size heterogeneity in the combined male-female sample. Full output is provided in **Supplementary Table 4**.

### 3.8 Sensitivity analysis with alternative outcome definitions

To assess the robustness of findings to the binary outcome classification, three pre-specified sensitivity analyses with alternative outcome definitions were performed. First, only complete remission (euthyroidism without levothyroxine replacement) was classified as treatment success; thyroid weight remained a significant predictor (adjusted OR = 1.018, 95% CI: 1.005-1.031, P = 0.005), though the RCS non-linearity was not significant under this definition (P-nonlinear=0.983). Second, among the 362 patients who achieved remission, thyroid weight did not discriminate between complete remission and hypothyroidism (OR = 1.000, P = 0.987), providing empirical justification for combining these outcomes. Third, when hypothyroidism was excluded entirely (complete remission vs non-remission, n=321), thyroid weight remained a strong predictor (adjusted OR = 1.031, 95% CI: 1.016-1.046, P<0.001), with non-significant non-linearity (P-nonlinear=0.177). Collectively, these analyses demonstrate that the thyroid weight-outcome association is robust across alternative outcome definitions, while the non-linear pattern is partially dependent on the inclusion of hypothyroidism as treatment success. The 46-g threshold should therefore be interpreted as exploratory. Full results are provided in **Supplementary Table 5**.

## 4 Discussion

This multi-center study provides critical insights into the relationship between thyroid weight and the efficacy of I-131 therapy in GD. Our analysis reveals a significant, non-linear, and threshold-dependent association characterized by an exploratory inflection point at approximately 46 grams. The principal finding is that the risk of non-remission increases continuously across the thyroid weight spectrum, with both segments below and above 46 g showing significant per-gram risk increases and overlapping confidence intervals, suggesting a continuous rather than discretely thresholded dose–effect relationship.

The observed non-linear relationship between thyroid weight and I-131 treatment outcome underscores the complex physiological and radiobiological processes involved in Graves’ disease therapy. The significant per-gram increase in the odds of non-remission across the entire thyroid weight range indicates that gland size is a continuous risk factor for treatment failure, with each additional gram of thyroid tissue conferring a consistent incremental risk. This could be attributed to several mechanisms. Smaller glands may exhibit a different metabolic profile or a more uniform distribution of I-131, meaning that a given administered activity achieves a more potent cytotoxic effect per gram of tissue. Conversely, an increase in volume, even if slight, might rapidly dilute the radioiodine concentration, reducing the effective absorbed dose to a sub-therapeutic level more dramatically than in larger glands where the initial concentration is already lower or more heterogeneously distributed (16, 17). The finding that increased early RAIU3h predicts higher odds of non-remission further supports the notion that rapidly metabolizing forms of hyperthyroidism are more challenging to treat (17). Rapid iodine turnover, often indicated by higher early uptake, can lead to a shorter residence time of I-131 in the thyroid, thereby reducing the delivered radiation dose and contributing to therapeutic failure (18). Additionally, a disease duration exceeding two years was associated with a two-fold increased odds of non-remission, possibly indicating more entrenched pathological changes or an advanced disease state less responsive to therapy.

Above 46 g, the incremental risk of non-remission per gram of thyroid tissue remains significant, with overlapping confidence intervals between the two segments suggesting a continuous dose–effect gradient rather than a discrete saturation point. The persistently elevated per-gram risk in larger glands may reflect several factors. Larger glands possess a greater total iodine pool, which can lead to a dilution of the administered radioiodine, resulting in a lower absorbed dose per unit of thyroid tissue (16). Furthermore, the architectural complexity of larger goiters, including nodule formation or fibrotic changes, might impede homogeneous radioiodine distribution, leading to areas that are relatively radio-resistant or under-dosed. Such glands may also develop compensatory mechanisms against the destructive effects of radiation, as suggested by dose-response curves for hypothyroidism (19). The absence of a significant association for IDPG in the multivariate model, despite its univariate significance, indicates that the current clinical practice of adjusting I-131 activity based on gland size (as reflected by IDPG) may already account for some of these effects, but it also underscores that thyroid weight itself maintains an independent predictive value beyond that accounted for by RAI uptake.

Our findings align with a substantial body of literature indicating that thyroid weight is a crucial determinant of I-131 therapy outcomes in GD (20). Numerous studies and meta-analyses have consistently reported an inverse correlation between thyroid volume and the success rate of I-131 treatment; larger glands are frequently associated with higher rates of treatment failure or the need for repeat doses (17). For instance, Shalaby et al. concluded in a meta-analysis that thyroid volume was positively associated with I-131 therapy failure (20). Similarly, Yu et al. identified iodine dose per gram of thyroid tissue as a significant independent predictor of I-131 efficacy, consistent with our study’s findings on the importance of an adequate absorbed dose (21).

However, a key novelty of our study lies in the precise quantitative characterization of the non-linear relationship and the identification of a specific inflection point at 46 g. While previous research acknowledged the complexity of dose-response relationships and the limitations of linear models, many did not employ advanced statistical techniques capable of identifying such thresholds. Studies often assumed linear correlations or relied on categorical analyses of thyroid size, which can obscure nuanced dose-response dynamics and lead to efficiency loss. For example, Mariotti highlighted that categorizing continuous exposures can be criticized when non-linear dose-response associations are expected (22). Our application of RCS offers a robust methodological advancement by allowing flexible modeling of continuous predictors without imposing restrictive parametric assumptions, thereby more accurately capturing the true biological relationship (23). The subsequent Piecewise Threshold Analysis (PTA) further validated the clinical relevance of the 46 g threshold, providing a more granular understanding than previously achieved. This approach refines predictive accuracy beyond traditional logistic regression, which might either under- or overestimate risk at different points along the thyroid weight spectrum if non-linearity is not accounted for (24). The identified threshold contributes significantly to filling a knowledge gap regarding the specific points at which the impact of thyroid size on I-131 efficacy undergoes a distinct change.

This study holds substantial academic value by advancing the theoretical understanding of dose-response relationships in radionuclide therapy. It demonstrates that complex biological phenomena often necessitate sophisticated statistical modeling beyond linear assumptions, offering a methodological blueprint for future research in personalized medicine (22, 25). The use of RCS and PTA to precisely delineate a threshold effect for thyroid weight contributes to refining predictive models for I-131 treatment outcomes in GD, moving towards a more data-driven approach to patient management (24).

From a practical perspective, these findings have important clinical applications. The identification of a 46 g threshold for thyroid weight can guide more individualized I-131 dosing strategies, particularly in patients with gland sizes close to this inflection point. For individuals across the thyroid weight spectrum, the continuous dose–effect relationship supports meticulous, individualized dose calculation. The 46 g threshold may serve as an exploratory reference for risk stratification, although the overlapping confidence intervals of the two segments suggest that dosing decisions should not rely on this threshold alone. Prospective validation is needed before clinical implementation. ​This refined understanding facilitates improved risk stratification and patient counseling, enabling more informed discussions about expected treatment outcomes and potential complications (16). Ultimately, optimizing dosing based on this non-linear relationship has the potential to enhance therapeutic success rates, reduce instances of treatment failure, and minimize adverse effects such as post-treatment hypothyroidism, thereby improving patient quality of life and healthcare efficiency (26).

The study was conducted in Guangxi Province, southwestern China, a region selected for its informative iodine nutritional landscape. Guangxi encompasses heterogeneous iodine environments ranging from historically iodine-deficient mountainous areas to iodine-adequate coastal regions. This diversity in population iodine exposure provides natural variability in thyroid size at baseline, strengthening the dose-response analysis. The two participating centers are the primary referral centers for RAI therapy in northern and central Guangxi, respectively, jointly managing approximately 400–500 new GD patients receiving RAI therapy annually. Both centers follow the Chinese Society of Nuclear Medicine (CSNM) guidelines for individualized RAI dose calculation, ensuring standardized treatment protocols. The baseline characteristics of our cohort (mean age 41 years; 73.9% female; median thyroid weight 46.5 g) are comparable to published GD cohorts from other Chinese regions and international populations, supporting the generalizability of the findings.

Several limitations of this study should be acknowledged. First, its retrospective design inherently introduces the potential for selection bias and residual confounding from unmeasured variables, which cannot be entirely eliminated despite DAG-based covariate adjustment. Second, thyroid weight was estimated using planar scintigraphy with ^99m^TcO_4_^-^; while widely used in clinical practice, this method may differ in precision from volumetric assessment by ultrasound or SPECT/CT. Third, study center was modeled as a fixed-effect covariate; a center-stratified analysis or generalized linear mixed model with center as a random effect may better capture institutional heterogeneity in equipment, treatment protocols, and patient characteristics. Fourth, although formal mediation analysis demonstrated that RAIU at 3 h accounts for only approximately 5.8% of the observed association (Sobel Z = 2.43, P = 0.015), residual mediation through unmeasured dose-related pathways cannot be fully excluded. Future studies incorporating SPECT/CT-based 3D dosimetry and formal causal mediation analysis with multiple mediators would be valuable for disentangling these pathways. Fifth, the study focused on clinical remission outcomes without examining long-term quality-of-life metrics or Graves’ ophthalmopathy progression. Sixth, sensitivity analyses demonstrated that the observed non-linear association is partially dependent on the inclusion of hypothyroidism in the treatment success category; the 46-g threshold should therefore be interpreted as exploratory and requires external validation. Seventh, the non-linearity was not confirmed in the female subgroup (P-nonlinear = 0.813), suggesting that the threshold effect may vary by sex, a finding that warrants investigation in larger, sex-stratified cohorts. Eighth, the mediation analysis examined only a single mediator (RAIU3h); the thyroid weight–outcome association may operate through multiple pathways including RAIU24h, effective half-life, and intra-thyroidal dose heterogeneity, and the total mediated effect may therefore be underestimated. Future studies employing multiple-mediator causal mediation analysis (VanderWeele & Vansteelandt, 2014) and SPECT/CT-based voxel dosimetry would be valuable for partitioning the indirect effect. Ninth, treatment outcomes were assessed within 12 months post-therapy; delayed hypothyroidism or late remission occurring beyond this window may result in outcome misclassification (right-censoring bias), potentially attenuating or inflating the observed thyroid weight effect. To address these limitations, future research should prioritize prospective, multicenter trials with standardized imaging protocols. Integrating SPECT/CT-based 3D dosimetry with thyroid volumetry could provide more accurate individualized absorbed dose estimates. Prospective evaluation of the 46 g threshold in individualized dosing algorithms is needed to translate these findings into evidence-based clinical guidelines

## 5 Conclusion

In conclusion, this study characterizes a novel non-linear, threshold-dependent relationship between thyroid weight and I-131 treatment efficacy in GD, identifying an exploratory inflection point at approximately 46 g of thyroid tissue. This finding significantly refines our understanding of how gland size influences therapeutic outcomes, advocating for a personalized approach to I-131 dosing. Future research should focus on validating this threshold in broader, diverse cohorts and exploring the underlying mechanistic determinants of thyroid radiosensitivity to further enhance the precision-based management of GD.

## Data Availability

The original contributions presented in the study are included in the article/**Supplementary Material**. Further inquiries can be directed to the corresponding author.
