# Supplementary figures and images for: Non-linear association between thyroid scintigraphy-derived thyroid weight and I-131 treatment efficacy in graves’ disease: a multicenter restricted cubic spline and threshold analysis
Source: Front Endocrinol (Lausanne). 2026 Jul 17;17:1802163. doi: 10.3389/fendo.2026.1802163 (PMC13423633; doi:10.3389/fendo.2026.1802163)

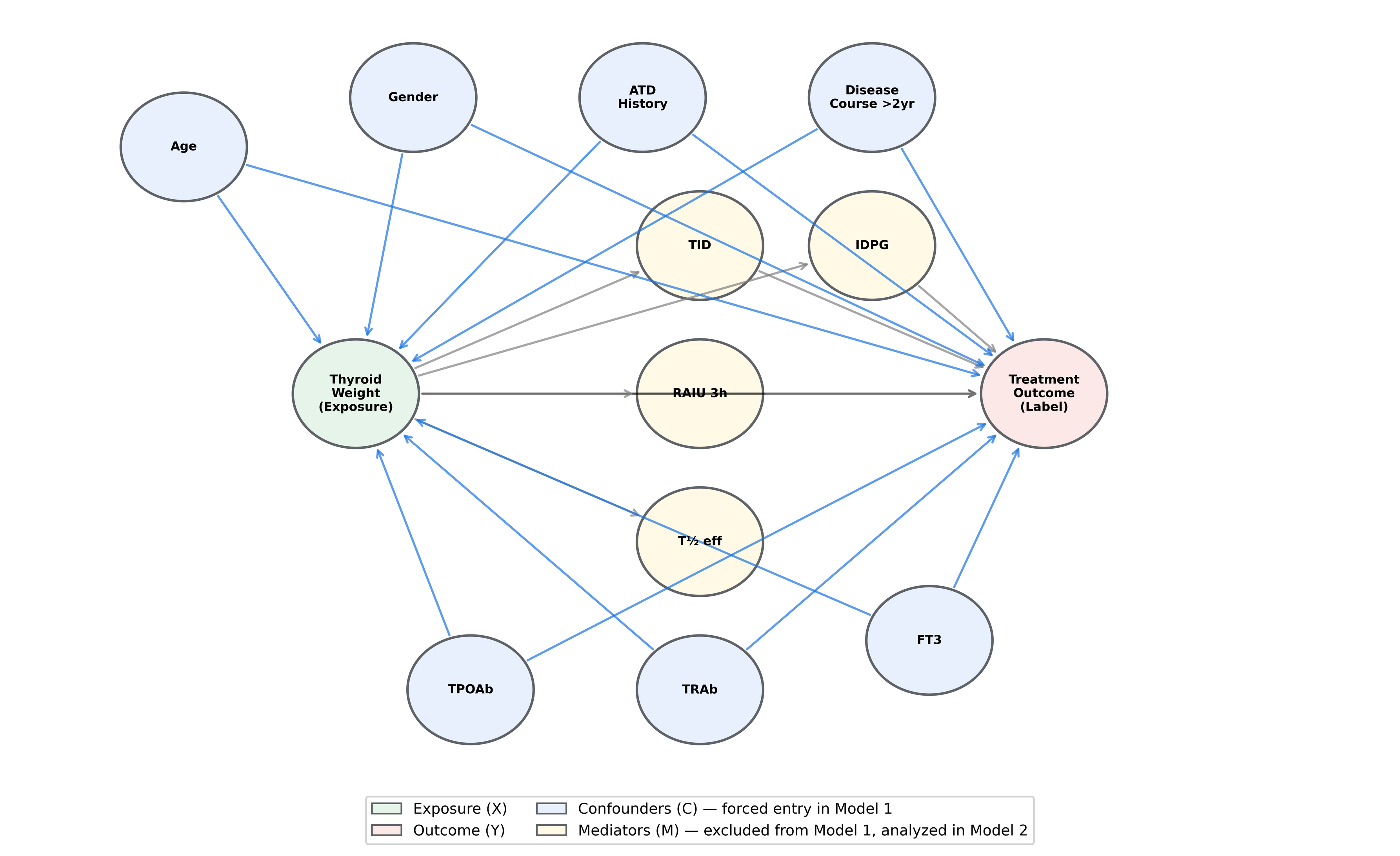

Supplement: Supplementary file 1 [file Image1.tiff]

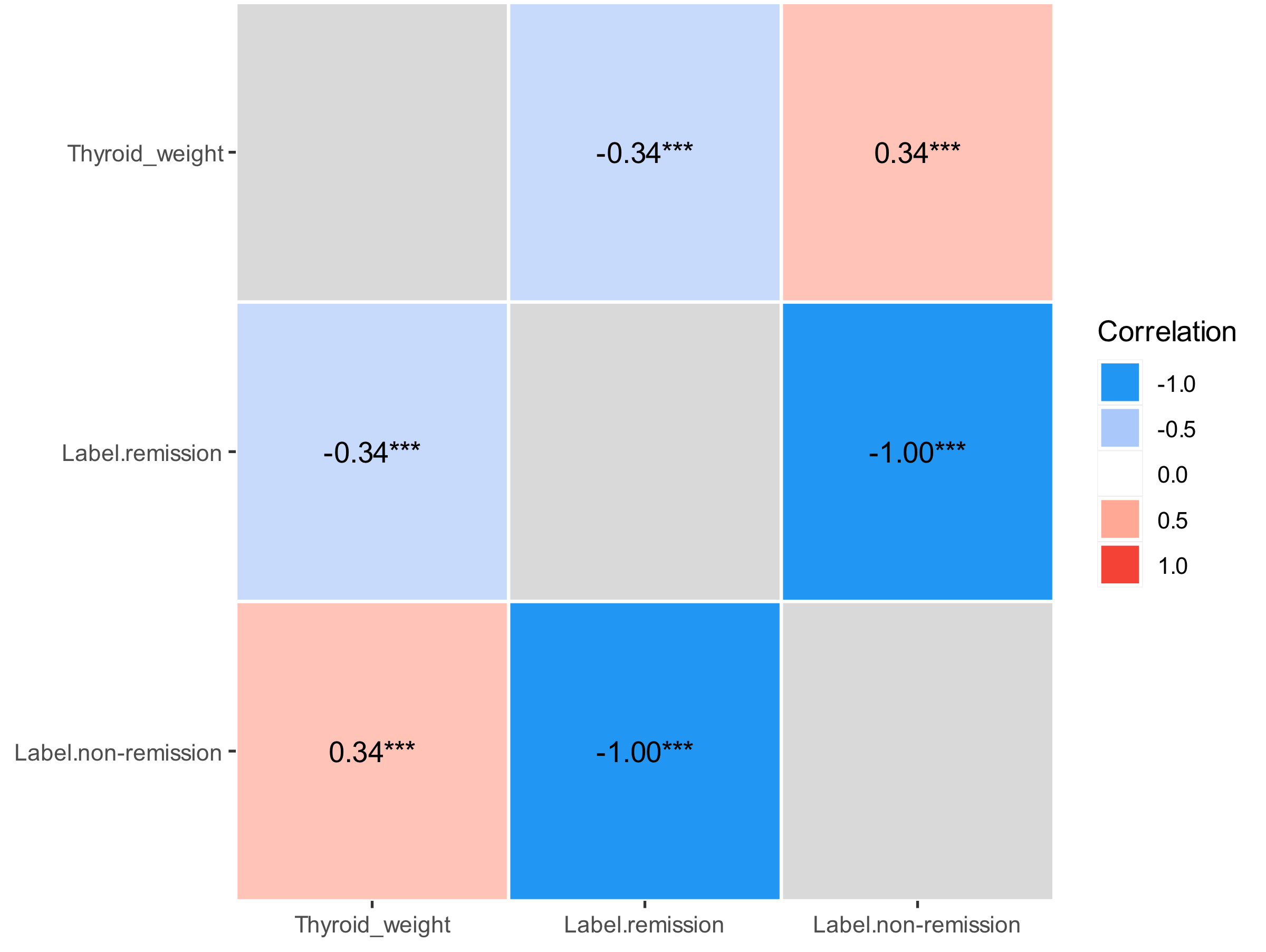

Supplement: Supplementary file 2 [file Image2.tiff]
