# Supplementary material for: Non-linear association between thyroid scintigraphy-derived thyroid weight and I-131 treatment efficacy in graves’ disease: a multicenter restricted cubic spline and threshold analysis
Source: Front Endocrinol (Lausanne). 2026 Jul 17;17:1802163. doi: 10.3389/fendo.2026.1802163 (PMC13423633; doi:10.3389/fendo.2026.1802163)
Supplement: Supplementary file 3 [file Table1.docx]

**Supplementary Materials**

**Supplementary Table S1. Variable definitions and coding scheme.**

| **Variable** | **Type** | **Unit / Coding** | **Role in Model** |
| --- | --- | --- | --- |
| Thyroid weight | Continuous | Per 1-g increase (g) | Exposure (fixed) |
| Age | Continuous | Years | Confounder (DAG) |
| Gender | Binary | 0 = Male; 1 = Female | Confounder (DAG) |
| ATD history | Binary | 0 = No; 1 = Yes | Confounder (DAG) |
| Disease course >2 years | Binary | 0 = ≤2 years; 1 = >2 years | Confounder (DAG) |
| FT3 | Continuous | pmol/L | Confounder (DAG) |
| TPOAB | Continuous | IU/mL | Confounder (DAG) |
| TRAB | Continuous | IU/L | Confounder (DAG) |
| TID | Continuous | Total I-131 dose (mCi) | Mediator (excluded) |
| IDPG | Categorical | 70–90 μCi/g; 91–120 μCi/g | Mediator (excluded) |
| RAIU at 3 h | Continuous | % | Mediator (excluded) |
| RAIU at 24 h | Continuous | % | Mediator (excluded); collinear with RAIU3h (r = 0.78) |
| Teff | Continuous | Effective half-life (days) | Mediator (excluded); collinear with RAIU3h (r = −0.66) |
| FT4 | Continuous | pmol/L | Excluded; collinear with FT3 (r = 0.85) |
| Treatment outcome | Binary | 0 = Remission; 1 = Non-remission | Dependent variable |

*DAG = Directed Acyclic Graph. Variables classified as confounders were forced into all multivariable models (Model 1, total effect) regardless of univariate P-values. Variables classified as mediators were excluded from the primary model to avoid overadjustment bias. Excluded variables were removed due to high collinearity (Pearson r > 0.75). Abbreviations: ATD = antithyroid drug; TID = total iodine-131 dose; IDPG = iodine dose per gram of thyroid tissue; RAIU = radioactive iodine uptake; Teff = effective half-life; FT3 = free triiodothyronine; FT4 = free thyroxine; TPOAB = thyroid peroxidase antibody; TRAB = TSH receptor antibody.*

**Supplementary Materials**

**Supplementary Table S2. Multicollinearity diagnostics and correlation matrix.**

Panel A: Variance inflation factors (VIF) — full candidate variable set.

| **Variable** | **VIF (full model)** | **Assessment** |
| --- | --- | --- |
| Thyroid_weight | 4.34 | Acceptable |
| Age | 1.21 | Acceptable |
| the_history_of_ATD_usage | 1.26 | Acceptable |
| Disease_course_over_2_years | 1.25 | Acceptable |
| TPOAB | 1.11 | Acceptable |
| TRAB | 1.33 | Acceptable |
| FT3 | 4.56 | Acceptable |
| FT4 | 4.23 | Acceptable |
| RAIU3h | 32.94 | Severe (VIF > 10) |
| RAIU24h | 19.81 | Severe (VIF > 10) |
| Teff | 12.47 | Severe (VIF > 10) |
| TID | 4.32 | Acceptable |
| IDPG | 1.56 | Acceptable |

Panel B: VIF — DAG-based Models 1 and 2.

| **Variable** | **Model 1 VIF (DAG)** | **Model 2 VIF (+RAIU3h)** |
| --- | --- | --- |
| Thyroid_weight | 1.24 | 1.32 |
| Age | 1.13 | 1.19 |
| Gender | 1.04 | 1.08 |
| the_history_of_ATD_usage | 1.19 | 1.24 |
| Disease_course_over_2_years | 1.21 | 1.22 |
| FT3 | 1.24 | 1.30 |
| TPOAB | 1.09 | 1.10 |
| TRAB | 1.23 | 1.28 |
| RAIU3h | — | 1.51 |
| Study center | 1.08 | 1.12 |

Panel C: Key pairwise Pearson correlation coefficients.

| **Variable pair** | **Pearson's r** |
| --- | --- |
| FT3 vs FT4 | 0.85 |
| RAIU3h vs RAIU24h | 0.78 |
| RAIU3h vs Teff | -0.66 |
| Thyroid weight vs Age | -0.28 |
| RAIU3h vs Thyroid weight | 0.35 |
| Thyroid weight vs TID | 0.67 |
| Thyroid weight vs IDPG | -0.27 |

*VIF = variance inflation factor. VIF > 5 indicates moderate collinearity; VIF > 10 indicates severe collinearity. The full candidate model (13 predictors) exhibited severe multicollinearity driven by RAIU3h (VIF = 32.9), RAIU24h (VIF = 19.8), and Teff (VIF = 12.5). In the DAG-based Model 1 (8 terms), all VIF values were < 1.25, indicating negligible collinearity. Addition of RAIU3h in Model 2 increased the maximum VIF to 1.82, which remains acceptable.*

**Supplementary Table S3. Change-in-estimate analysis for confounder selection.**

| **Variable added to Thyroid_weight** | **Adjusted OR for Thyroid_weight** | **95% CI** | **OR change from baseline** | **≥10% change?** |
| --- | --- | --- | --- | --- |
| Age | 1.0290 | (1.0213, 1.0367) | 0.2% | No |
| Gender | 1.0304 | (1.0228, 1.0380) | 5.2% | No |
| ATD history | 1.0274 | (1.0200, 1.0349) | 5.2% | No |
| Disease >2 yr | 1.0268 | (1.0194, 1.0343) | 7.2% | No |
| FT3 | 1.0295 | (1.0220, 1.0371) | 2.1% | No |
| TPOAB | 1.0290 | (1.0215, 1.0365) | 0.3% | No |
| TRAB | 1.0282 | (1.0208, 1.0357) | 2.3% | No |
| RAIU3h (mediator) | 1.0237 | (1.0160, 1.0314) | 18.1% | Yes |

*Baseline OR (Thyroid_weight only): 1.0289 (95% CI: 1.0216, 1.0363), P < 0.001. OR change = |OR_adjusted − OR_baseline| / |OR_baseline − 1| × 100%. A change of ≥10% was pre-specified as the threshold for considering a variable an important confounder. No individual covariate exceeded this threshold (range: 0.2%–7.2%), confirming the stability of the DAG-based confounder set.*

**Supplementary Table S4. Female subgroup sensitivity analysis (n = 452).**

**Panel A: DAG-adjusted logistic regression (Model 1, excluding gender).**

| **Variable** | **Adjusted OR** | **95% CI** | **P-value** |
| --- | --- | --- | --- |
| Intercept | 0.076 | (0.022, 0.260) | <0.001 *** |
| Thyroid weight (per 1 g) | 1.041 | (1.028, 1.053) | <0.001 *** |
| Age (per year) | 1.013 | (0.995, 1.032) | 0.162 |
| ATD history (Yes vs No) | 1.114 | (0.647, 1.917) | 0.698 |
| Disease course >2 yr (vs ≤2 yr) | 1.512 | (0.909, 2.515) | 0.111 |
| FT3 (per pmol/L) | 0.997 | (0.978, 1.017) | 0.788 |
| TPOAB (per IU/mL) | 1.000 | (0.999, 1.000) | 0.193 |
| TRAB (per IU/L) | 1.002 | (0.982, 1.023) | 0.820 |
| Study center (External vs Guilin) | 0.482 | (0.297, 0.782) | 0.003 ** |

Panel B: Restricted cubic spline analysis in female subgroup (n = 452).

| **Parameter** | **Value** |
| --- | --- |
| Knot positions (percentiles) | 10th = 26.0 g; 50th = 44.6 g; 90th = 90.6 g |
| TW_linear coefficient | 0.052589 |
| TW_nonlinear coefficient | -0.000004 |
| P-nonlinear (Wald test, df = 1) | 0.813* |
| LRT: RCS vs linear (χ², P) | χ² = 0.06, P = 0.814* |
| AIC: linear model | 521.3 |
| AIC: RCS model | 523.3 |
| ΔAIC (RCS − linear) | +2.0 |

Panel C: Piecewise threshold analysis in female subgroup (break-point = 46 g).

| **Parameter** | **Value** |
| --- | --- |
| Standard linear OR (95% CI) | 1.041 |
| TW < 46 g: OR (95% CI) | 1.050 (1.013, 1.089) |
| TW ≥ 46 g: OR (95% CI) | 1.089 (1.048, 1.131) |
| LRT: piecewise vs linear (χ², P) | χ² = 0.35, P = 0.555 |
| AIC: linear | 521.3 |
| AIC: piecewise | 522.9 |

*Panel D: Model fit summary. Female cohort: N = 452, remission events = 260, non-remission events = 192. Linear model AUC = 0.712 (calculated as accuracy). Model 1 AIC = 521.3, log-likelihood = -250.6.*

**Supplementary Table S5. Sensitivity analysis with alternative outcome definitions.**

**Panel A: Primary Analysis — Complete Remission + Hypothyroidism vs Partial Remission + Ineffective (N = 612)**

*Events/Total: 250/612; AUC = 0.702; AIC = 743.3*

| **Variable** | **OR (95% CI)** | **P-value** |  |
| --- | --- | --- | --- |
| Intercept | 0.095 (0.033–0.272) | 0.0000 | *** |
| Thyroid weight (per 1 g) | 1.025 (1.017–1.034) | <0.001 | *** |
| Age (per year) | 1.002 (0.988–1.017) | 0.7479 |  |
| FT3 (per pmol/L) | 0.993 (0.978–1.009) | 0.4065 |  |
| TPOAB (per IU/mL) | 1.000 (0.999–1.000) | 0.1969 |  |
| TRAB (per IU/L) | 1.015 (0.999–1.032) | 0.0705 |  |
| Center (External vs Guilin) | 0.624 (0.417–0.934) | 0.022 | * |

**Panel B: Sensitivity 1 — Complete Remission vs All Others (N = 612)**

*Events/Total: 541/612; AUC = 0.868; AIC = 475.7*

| **Variable** | **OR (95% CI)** | **P-value** |  |
| --- | --- | --- | --- |
| Intercept | 0.889 (0.218–3.619) | 0.8696 |  |
| Thyroid weight (per 1 g) | 1.018 (1.005–1.031) | 0.0051 | ** |
| Age (per year) | 1.000 (0.981–1.020) | 0.9833 |  |
| FT3 (per pmol/L) | 1.006 (0.983–1.029) | 0.6264 |  |
| TPOAB (per IU/mL) | 1.000 (1.000–1.001) | 0.4936 |  |
| TRAB (per IU/L) | 1.014 (0.991–1.038) | 0.2251 |  |
| Center (External vs Guilin) | 2.585 (1.484–4.503) | 0.0008 | *** |

**Panel C: Sensitivity 2 — Complete Remission vs Hypothyroidism Among Remission Patients (N = 362)**

*Events/Total: 280/362; AUC = 0.768; AIC = 380.9*

| **Variable** | **OR (95% CI)** | **P-value** |  |
| --- | --- | --- | --- |
| Intercept | 1.482 (0.304–7.224) | 0.6264 |  |
| Thyroid weight (per 1 g) | 1.000 (0.987–1.014) | 0.9866 |  |
| Age (per year) | 0.996 (0.975–1.017) | 0.6945 |  |
| FT3 (per pmol/L) | 1.014 (0.990–1.040) | 0.2577 |  |
| TPOAB (per IU/mL) | 1.000 (1.000–1.001) | 0.3317 |  |
| TRAB (per IU/L) | 1.008 (0.983–1.034) | 0.5242 |  |
| Center (External vs Guilin) | 3.379 (1.901–6.008) | 0.0000 | *** |

**Panel D: Sensitivity 3 — Complete Remission vs Non-Remission, Excluding Hypothyroidism (N = 321)**

*Events/Total: 250/321; AUC = 0.788; AIC = 345.5*

| **Variable** | **OR (95% CI)** | **P-value** |  |
| --- | --- | --- | --- |
| Intercept | 0.154 (0.030–0.789) | 0.0249 | * |
| Thyroid weight (per 1 g) | 1.031 (1.016–1.046) | 0.0000 | *** |
| Age (per year) | 0.997 (0.974–1.020) | 0.7755 |  |
| FT3 (per pmol/L) | 1.003 (0.978–1.029) | 0.7944 |  |
| TPOAB (per IU/mL) | 1.000 (0.999–1.001) | 0.9111 |  |
| TRAB (per IU/L) | 1.027 (1.000–1.054) | 0.0486 | * |
| Center (External vs Guilin) | 1.748 (0.913–3.345) | 0.0918 |  |

**Panel E: RCS Non-linearity Tests Across Outcome Definitions**

| **Outcome Definition** | **N** | **P-nonlinear (Wald)** | **LRT P-value** |
| --- | --- | --- | --- |
| CR+Hypo vs PR+Ineff (Primary) | 612 | 0.0286 | 0.0328 |
| CR vs All Others (S1) | 612 | 0.9828 | 0.9828 |
| CR vs PR+Ineff, no Hypo (S3) | 321 | 0.1771 | 0.2116 |
